# Deaf and non-deaf basketball and volleyball players' multi-faceted difference on repeated counter movement jump performances: Height, force and acceleration

**DOI:** 10.3389/fspor.2022.941629

**Published:** 2022-09-14

**Authors:** Recep Soslu, Ömer Özer, Abdullah Uysal, Ömer Pamuk

**Affiliations:** Sport Science Faculty, Karamanoǧlu Mehmetbey University, Karaman, Turkey

**Keywords:** basketball, volleyball, RCMJ, deaf, biomechanics, performance

## Abstract

The aim of this study was to compare the performances of Repetitive Counter Movement Jumping (basketball/volleyball) of deaf/non-deaf athletes. Athletes playing in the Turkish Deaf Basketball and Volleyball national teams and in Basketball and Volleyball First Leagues participated in the study. The study group consisted of 51 male athletes, including deaf basketball (*n* = 11; age: 26.18 ± 4.79 years), deaf volleyball (*n* = 12, age: 26.33 ± 4.27 years), non-deaf basketball (*n* = 14, age: 26.93 ± 4.87 years), and non-deaf volleyball (*n* = 14, age: 24.93 ± 5.10 years) players. As a result of the test, Jump Height from Take Off Velocity, Jump Height from Take Off Velocity, Jump Height from Flight Time, Counter Movement Acceleration, Push Off Acceleration, Average Velocity, Average Power, and Average Force were analyzed. Differences in the jump performances among the groups (deaf basketball and volleyball, non-deaf basketball, and volleyball) were tested by one-way analyses of variance (ANOVA) with Tukey's *post-hoc* follow-up testing when necessary for jump test. As a result, this is the first study to investigate the number of jumps and jump height, the force produced, acceleration at the time of jump, and jump velocity during 30 s in deaf and non-deaf basketball and volleyball players within the scope of individual Repeated Counter Movement Jump test. Based on the biomechanical changes according to our results, our findings show a greater decrease in the number of jumps and jump heights, the force produced, the acceleration at the moment of the jump and the jump velocity in all athletes, especially those that affect the deaf.

## Introduction

The term “deaf” is defined by the International Sports Committee for the Deaf as the perception of sound only at 55 dB or more through the ear that hears better (Jordan, 2001; Schilder et al., 2017). Deaf athletes can compete in sports competitions without significant restrictions owing to their physical strength. The main difference between them and healthy athletes is obstacles to communication (Kurková et al., 2011). Problems occurring in muscle control depending on how much the auditory center is affected negatively influence muscle strength, motor functions, and jump performance (Lieberman et al., 2004; Schwab and Kontorinis, 2011). In the literature, it has been reported that the physical abilities of healthy individuals are better than those of deaf individuals (Kurkova, 2009; Kurkova et al., 2010). Therefore, chronic exercises are important for improved physical fitness and ability performance of deaf athletes. This situation should also be well-assessed in terms of performance in branches such as basketball and volleyball, where biomotoric characteristics are used at a high level. Basketball is a sports branch where motoric characteristics such as directional change, speed and repetitive jump (vertical/horizontal) are used at a high level during competitions or training (Bhadu and Singh, 2016), whereas volleyball is a sports branch that includes performance parameters such as jump, block, counter movement jump (CMJ), power, strength, agility, and speed in defensive and offensive actions (Weldon et al., 2021). Therefore, volleyball and basketball branches should be taken into account when evaluating the performance parameters of deaf and non-deaf athletes.

Repetitive Counter Movement Jump (RCMJ) is a vertical jump, mainly including cyclic eccentric/concentric muscle movement that involves muscle movements of the trunk, hip, knee, and ankle extensors. RCMJ is a vertically successive jump performance parameter in which characteristics such as joint range of motion (ROM), strength and flexibility of muscles during movement are at the forefront (Ashley and Weiss, 1994; Markovic, 2007). During the jump, we can be analyze the basic biomechanical and functional characteristics of the neuromuscular system (i.e., endurance, fatigue, elasticity, strength, etc.). For a perfect performance, it is important that the repeated jumps are as high as possible and in the shortest contact with the ground (McBride et al., 2008; Sattler et al., 2015). In volleyball and basketball, jumps such as block, spike, and rebound, which often need to be repeated, are widely used in both defensive and offensive actions (Kenny and Gregory, 2006). Therefore, the athlete needs to be able to maximize the jump height and minimize the time it takes to reach the maximum height (Masanori et al., 2021). Reactive force evaluates the ability to reach maximum jump height and minimum ground contact time during repetitive jump (Nariai et al., 2017). RCMJ test is an effective method used to assess performance parameters, such as Jump Height from Take Off Velocity, Jump Height from Flight Time, Counter Movement Acceleration, Push Off Acceleration, Velocity, Power, and Force. It is a fact that the strength of the lower extremity muscles (hip, leg, and ankle) is extremely important in RCMJs, particularly at the time of jumping (Deniskina and Levik, 2001; Oxfeldt et al., 2019). Therefore, strategies that optimize the ability to RCMJs should be a part of training programs (Sheppard et al., 2007). Many studies emphasize the importance of training programs developed for athletes' jump performances (Slimani et al., 2016; Stojanović et al., 2017). Although there are many studies in the literature investigating the relationship between the biomechanics of CMJ and performance (Szulc et al., 2017; Neuls et al., 2019), the number of studies using the Repetitive Counter Movement Jump (RCMJ) test is quite limited. In a presented study, there were statistically significant differences between deaf and hearing female soccer players, although the effect size was generally small in the biomechanical parameter (strength, power, H/Q, and jump height, respectively) (*p* < 0.05). In another study, mean values of CMJ were higher in the control group than in deaf football players, but the difference was not statistically significant (*p* = 0.07). Based on this, it was planned to determine the effect of neuromuscular fatigue on an important performance component of the repetitive jumps of deaf and non-deaf basketball/volleyball players.

## Materials and methods

### Participants

**Figure 1:**
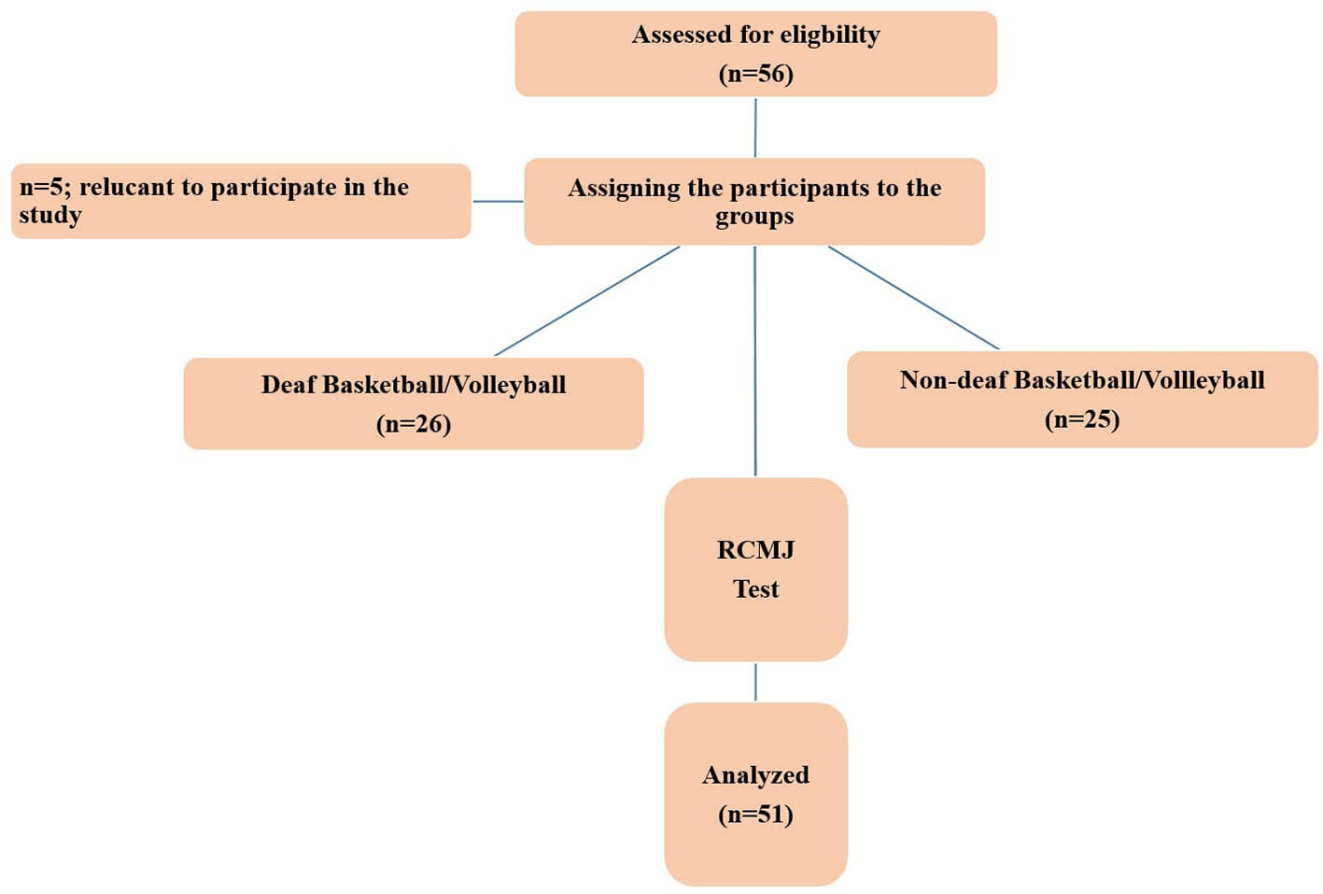
Study design.

Athletes playing in the Turkish Deaf Basketball and Volleyball national teams and in Basketball and Volleyball First Leagues participated in the study. The study group consisted of 51 male athletes, including deaf basketball (*n* = 11; height, body weight, age: 183.55 ± 10.51 cm, 83.89 ± 9.09 kg, and 26.18 ± 4.79 years, respectively), deaf volleyball (*n* = 12, height, body weight, age: 187.00 ± 7.07 cm, 80.47 ± 9.44 kg, and 26.33 ± 4.27 years, respectively), non-deaf basketball (*n* = 14, height, body weight, age: 196.93 ± 9.14 cm, 94.34 ± 8.60 kg, and 26.93 ± 4.87 years, respectively), and non-deaf volleyball (*n* = 14, height, body weight, age: 194.5 ± 5.02 cm, 86.27 ± 8.64 kg, and 24.93 ± 5.10 years, respectively) players. In the study, an “Cross-Sectional” model was employed, including the application of the factor, the relationship of which would be measured, to the athletes under certain conditions and rules, the measurement of athletes' responses to the factor, comparison of the results obtained, and making a decision. Criteria for athlete selection included being older than 18 years of age and competing at an elite level, deaf athletes' having a medically diagnosed hearing impairment (>55 dB hearing in both ears without cochlear implantation) and being able to understand the basic instructions given. Furthermore, the anatomical structures of all athletes participating in the study were healthy, and they had no injury-induced medical and orthopedic problems in the lower/upper extremities. Some of the athletes were not included in the study because they did not want to participate in the study (*n* = 5) (Figure 1). The athletes included in the study group were informed about the study (risk/benefit), and their written consent was obtained in accordance with the Declaration of Helsinki. The study was approved by the Clinical Ethics Committee of Karamanoglu Mehmetbey University (Doc No: 09-2021/157).

### Instrumentation

#### Anthropometric variables

The body weights and heights of the athletes were measured with a stadiometer (SECA-Mod. 220, Seca GmbH&Co. KG, Hamburg, Germany).

### Repetitive counter movement jump test (RCMJ)

RCMJ is a VJ, mainly including cyclic eccentric/concentric muscle movement that involves muscle movements of the trunk, hip, knee, and ankle extensors. Jump is a squat-pushing combined with many coordinated joint movements of the lower extremity and trunk. RCMJ is the biomechanically and functionally lowest semi-squat position (knee <90° and trunk/hips in the flexed position). RCMJ is performed for 30 s in succession and continuously, reaching the highest point at each jump. A three dimensional Kistler force plate (Dimensions: 600 × 500 × 50 mm; Type 5691A; Kistler, Winterthur, Switzerland) was used as the gold standard and criterion device for the Repetitive Counter Movement Jump (RCMJ) test of the athletes. The force plate was firmly placed on the ground to measure vertical reaction forces (Range: 0–10 kN; sampling rate: 1,000 Hz, FIR-Savitzky Golay Filter) during RCMJ. The force plate was connected to a personal computer (HP, ProBook 450 G6), and calculation was made using the proprietary software (Kistler Measurement, Analysis and Reporting Software: MARS), the above-mentioned formula, and gravitational force (*g[m/(s*^∧^*2)]*) (Makaraci et al., 2021b).

### Procedure

**Figure 2:**
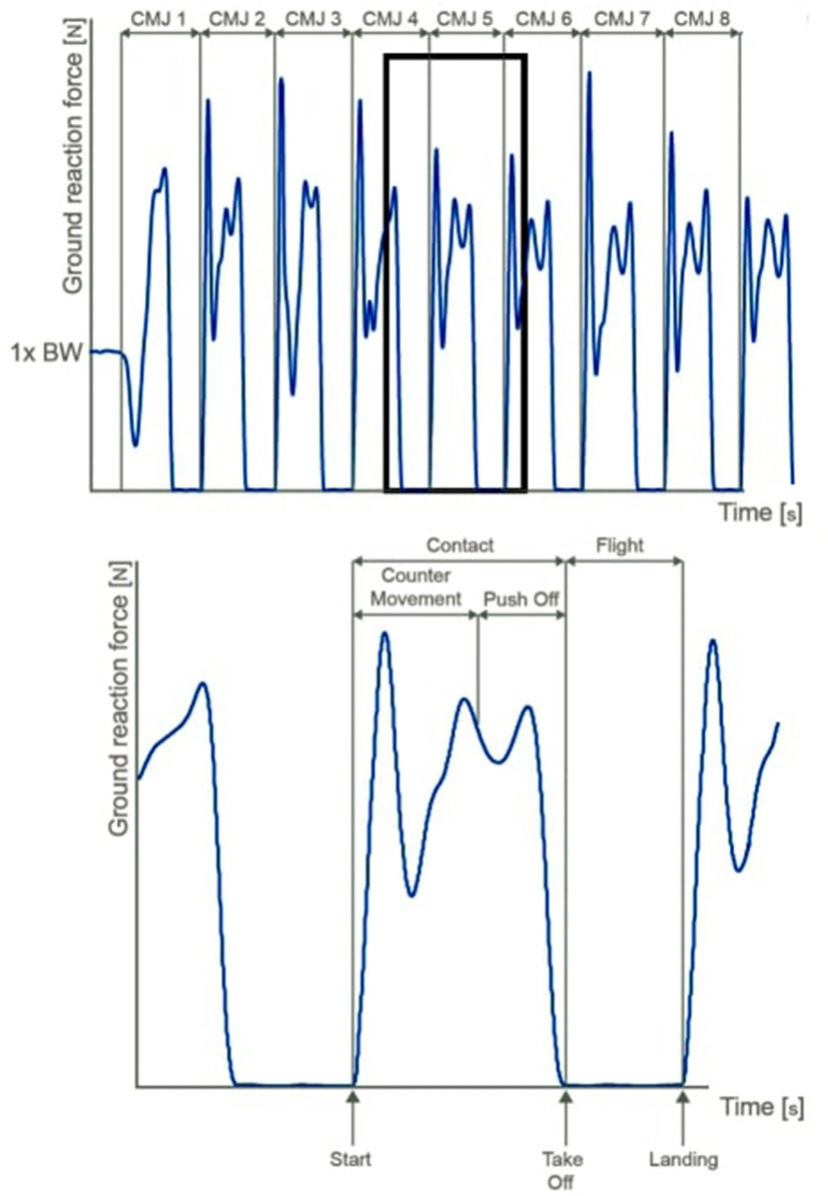
RCMJ analysis.

Before the application, the test protocol was explained by an expert translator for deaf athletes with the sign language and by an expert for healthy athletes. A trial was carried out, and possible errors were explained. After anthropometric measurements, a standard warm-up was performed, including a 5-min run, a 5-min passive stretching, and three maximum vertical jumps. The warm-up session was followed by a 5-min rest period (Theodorou et al., 2013). The athlete stood on the force plate with his feet shoulder-width apart, his hands on his waist, and his trunk upright. With the expert translator's (deaf/non-deaf) start command, the athlete performed the highest CMJ with knees <90° and in the bent position of the trunk/hips and then fell on the force plate. These successive jumps were performed for 30 s without stopping. The athlete was instructed to jump as high as possible in each cmj jump. Performance times of 30 s for each parameter in the test were divided into 3 sections (first, middle, and last) of 10 s. Within 30 s, the athlete's neuromuscular fatigue reveals the change in repetitive jumps in 10-s slices. If the athlete performed the technique incorrectly during the RCMJ test protocol, the protocol was stopped. The test was performed twice for each athlete. For the athlete's repeated test, the test was repeated for the second time with a 5-min passive recovery. The best value of the 2 tests was selected. All tests were performed between the same hours of the day (14:00–16:00) under standard environmental conditions (26 ± 2°C and 75 ± 4% relative humidity) and in the same order. As a result of the test, Jump Height from Take Off Velocity (JHTOV) (Total jumps over 30 s), Jump Height from Take Off Velocity [Average of first, middle, last n jumps and all jumps (m)], Jump Height from Flight Time (JHFT) [Average of first, middle, last n jumps and all jumps (m)], Counter Movement Acceleration (CMA) [Average of first, middle, last n jumps and all jumps (m/s^∧^2)], Push Off Acceleration (POA) [Average of first, middle, last n jumps and all jumps (m/s^∧^2)], Average Velocity (AV) [Average of first, middle, last n jumps and all jumps (m/s)], Average Power (AP) [Average of first, middle, last n jumps and all jumps (W)], and Average Force (AF) [Average of first, middle, last n jumps and all jumps (N)] were analyzed on the device's software (MARS) (Figure 2).

### Analyses

To establish sample size, a power analysis (priori) for a Cross-Sectional design was conducted using G^*^Power 3.1.6. Based on the effect sizes reported in comparable studies, the analysis indicated that minimally 14 participants for an α of 0.05 and a power of 0.95 would be required (Taylor, 2008). In data analysis, JHTOV, JHFT, CMA, POA, AV, AP, AF, and statistics of descriptive variables (age, height, and weight) were reported using mean, mean differences and standard deviation (mean ± SD). For each parameter, the 30-s performance times in the test were divided into 3 periods (first, middle, and last) of 10 s. The normality of the distribution was tested with Levene's test and skewness/kurtosis values. Differences in the jump performances (JHTOV, JHFT, CMA, POA, AV, AP, and AF) among the groups (deaf basketball and volleyball, non-deaf basketball and volleyball) were tested by one-way analyses of variance (ANOVA) with Bonferroni *post-hoc* follow-up testing when necessary for jump test. IBM SPSS 24.0 (IBM Co., Armonk, NY, USA) was used for statistical data analysis. To estimate effect sizes, eta squared (η2) was computed with η2 ≥ 0.01 indicating small, ≥0.06 medium and ≥0.14 large effects (Cohen, 1988). Statistical significance was set at α < 0.05.

## Results

At the end of the study, the statistics of the variables of “JHTOV, JHFT, CMA, POA, AV, AP, AF” regarding the RCMJ performances of deaf and non-deaf basketball/volleyball players were reported in tables as mean and standard deviation (mean ± SD).

Upon reviewing Table 1, a statistically significant difference was identified between the deaf and non-deaf basketball groups in terms of JHTOV, JHTOVC, and JHTOVD parameters. This difference was revealed to be statistically significant in favor of the non-deaf basketball group. In other parameters, it was found that there was a difference in favor of the non-deaf basketball group in general, but this difference was not statistically significant (*p* > 0.05). A statistically significant difference was observed between the deaf and non-deaf volleyball groups in terms of JHTOV, JHTOVA, JHTOVB, JHTOVC, JHTOVD, JHFTA, JHFTB, and JHFTD parameters. This difference was determined to be significant in favor of the non-deaf volleyball group. In the JHFTC parameter, a difference was found in favor of the non-deaf volleyball group, but this difference was not statistically significant (*p* > 0.05).

**Table 1:** Comparison of jump height from take off velocity and jump height from flight time of deaf and non-deaf basketball/volleyball players.

**Parameter's**	**Deafness basketball (n:11)**	**Basketball (n:14)**	***md***	***p***	***d***	**Deafness volleyball (n:12)**	**Volleyball (n:14)**	***md***	***p***	***d***
	**X ±SS**	**X ±SS**				**X ±SS**	**X ±SS**			
JHTOV (total jumps over 30 s)	21.04 ± 4.54	23.36 ± 4.78	−2.32	*P* = 0.19	0.50	17.42 ± 4.66	19.71 ± 3.60	−2.29	*P* = 0.19	0.55
JHTOVA (0–10 s) [m]	0.24 ± 0.09	0.28 ± 0.08	−0.04	*P* = 0.19	0.47	0.32 ± 0.09	0.35 ± 0.06	−0.03	*P* = 0.27	0.39
JHTOVB (10–20 s) [m]	0.21 ± 0.05	0.23 ± 0.07	−0.02	*P* = 0.51	0.33	0.27 ± 0.04	0.32 ± 0.08*	−0.05	*P* = 0.05	0.79
JHTOVC (20–30 s) [m]	0.16 ± 0.06	0.20 ± 0.05	−0.04	*P* = 0.40	0.72	0.26 ± 0.16	0.31 ± 0.10	−0.05	*P* = 0.62	0.37
JHTOVD (0–30 s) [m]	0.21 ± 0.04	0.24 ± 0.06	−0.03	*P* = 0.20	0.59	0.26 ± 0.04	0.33 ± 0.07*	−0.07	*P* = 0.001	1.23
JHFTA (0–10 s) [m]	0.21 ± 0.06	0.23 ± 0.05	−0.02	*P* = 0.46	0.36	0.26 ± 0.07	0.29 ± 0.04	−0.03	*P* = 0.26	0.53
JHFTB (10–20 s) [m]	0.19 ± 0.05	0.20 ± 0.05	−0.01	*P* = 0.66	0.20	0.25 ± 0.05	0.28 ± 0.05	−0.03	*P* = 0.04	0.60
JHFTC (20–30 s) [m]	0.17 ± 0.03	0.18 ± 0.04	−0.01	*P* = 0.62	0.28	0.20 ± 0.06	0.27 ± 0.05	−0.07	*P* = 0.008	1.27
JHFTD (0–30 s) [m]	0.20 ± 0.03	0.19 ± 0.23	0.01	*P* = 0.93	0.06	0.23 ± 0.05	0.29 ± 0.04	−0.06	*P* = 0.002	1.32

*P < 0.05. **md**, mean differences; **Cohen d** effect size where ≤ 0.2 = small, ≤ 0.5 = medium, and ≤ 0.8 = large.

When Table 2 was examined, a statistically significant difference was found between the deaf and non-deaf basketball groups in terms of CMAA, AVA, AVC, and AVD parameters (*p* < 0.05). A statistically significant difference was determined in AVA, AVC, and AVD parameters in favor of the deaf basketball group, and the difference in CMAA parameter was statistically significant in favor of the non-deaf basketball group. No statistically significant difference was observed in other parameters (*p* > 0.05). A statistically significant difference was revealed in terms of CMAA, CMAB, POAA, POAC, AVA, and AVD parameters between the deaf and non-deaf volleyball groups (*p* < 0.05). In CMAA, CMAB, POAA, AVA, and AVD parameters, there was a significant difference in favor of the non-deaf volleyball group and there was a statistically significant difference in POAC parameter in favor of the deaf volleyball group. No statistically significant difference was found in all other parameters (*p* > 0.05).

**Table 2:** Comparison of counter movement acceleration, push off acceleration and average velocity of deaf and non-deaf basketball/volleyball players.

**Parameter's**	**Deafness basketball (n:11)**	**Basketball (n:14)**	***md***	***p***	***d***	**Deafness volleyball (n:12)**	**Volleyball (n:14)**	***md***	***p***	***d***
	**X ±SS**	**X ±SS**				**X ±SS**	**X ±SS**			
CMAA (0–10 s) [m/s^∧^2]	6.80 ± 3.06	6.40 ± 2.11	0.40	*P* = 0.69	0.15	5.97 ± 2.57	5.23 ± 2.29	0.74	*P* = 0.45	0.30
CMAB (10–20 s) [m/s^∧^2]	6.24 ± 1.66	6.12 ± 2.03	0.12	*P* = 0.89	0.06	6.76 ± 2.46	6.10 ± 2.53	0.66	*P* = 0.48	0.26
CMAC (20–30 s) [m/s^∧^2]	6.47 ± 1.77	6.07 ± 2.48	0.40	*P* = 0.64	0.18	5.28 ± 1.62	5.39 ± 2.32	−0.11	*P* = 0.89	0.05
CMAD (0–30 s) [m/s^∧^2]	6.22 ± 1.98	6.02 ± 2.24	0.20	*P* = 0.82	0.09	5.72 ± 2.40	5.76 ± 2.19	−0.04	*P* = 0.72	0.02
POAA (0–10 s) [m/s^∧^2]	7.26 ± 2.16	7.16 ± 1.44	0.10	*P* = 0.91	0.05	7.09 ± 1.89	5.81 ± 2.56	1.28	*P* = 0.12	0.57
POAB (10–20 s) [m/s^∧^2]	6.28 ± 1.90	6.02 ± 1.39	0.26	*P* = 0.74	0.16	7.02 ± 2.26	6.22 ± 2.01	0.80	*P* = 0.29	0.37
POAC (20–30 s) [m/s^∧^2]	5.98 ± 1.13	6.00 ± 2.02	−0.02	*P* = 0.97	0.01	4.80 ± 1.84	5.33 ± 1.38	−0.53	*P* = 0.42	0.32
POAD (0–30 s) [m/s^∧^2]	6.01 ± 1.47	6.44 ± 1.63	−0.43	*P* = 0.49	0.28	6.30 ± 1.37	6.05 ± 1.73	0.25	*P* = 0.68	0.16
AVA (0–10 s) [m/s^∧^2]	1.22 ± 0.23*	1.34 ± 0.22	−0.12	*P* = 0.02	0.53	1.38 ± 0.21	0.97 ± 0.48*	0.41	*P* = 0.00	1.11
AVB (10–20 s) [m/s^∧^2]	1.12 ± 0.38	1.16 ± 0.42	−0.04	*P* = 0.10	0.09	1.37 ± 0.31	1.03 ± 0.47	0.34	*P* = 0.37	0.08
AVC (20–30 s) [m/s^∧^2]	1.15 ± 0.21*	1.20 ± 0.23	−0.05	*P* = 0.05	0.23	1.12 ± 0.37	0.94 ± 0.39	0.18	*P* = 0.70	0.04
AVD (0–30 s) [m/s^∧^2]	1.18 ± 0.15*	1.24 ± 0.23	−0.06	*P* = 0.05	0.31	1.27 ± 0.17	1.01 ± 0.42*	0.26	*P* = 0.00	0.81

*P < 0.05; **md**, mean differences, **Cohen d** effect size where ≤ 0.2 = small, ≤ 0.5 = medium, and ≤ 0.8 = large.

Upon reviewing Table 3, there was a statistically significant difference between the deaf and non-deaf basketball groups in favor of the non-deaf basketball group in all parameters of APA, APB, APC, APD, AFA, AFB, AFC, and AFD. Statistically significant differences were seen between the deaf and non-deaf volleyball groups (*p* < 0.05). A statistically significant difference was found in favor of deaf volleyball players in APA, APB, and AFA parameters and there was a statistically significant difference in favor of non-deaf volleyball players in APC, AFC, and AFD parameters.

**Table 3:** Comparison of average power and force of deaf and non-deaf basketball/volleyball players.

**Parameter's**	**Deafness basketball (n:11)**	**Basketball (n:14)**	***md***	***p***	***d***	**Deafness volleyball (n:12)**	**Volleyball (n:14)**	***md***	***p***	***d***
	**X ±SS**	**X ±SS**				**X ±SS**	**X ±SS**			
APA (0–10 s) [W]	1618.96 ± 404.60	2028.14 ± 349.69	−409.18	*P* = 0.13	1.08	1787.17 ± 359.55*	1580.69 ± 609.01	206.487	*P* = 0.009	0.41
APB (10–20 s) [W]	1412.81 ± 543.25	1742.47 ± 420.25	−329.66	*P* = 0.13	0.68	1812.75 ± 420.87*	1638.71 ± 560.35	174.08	*P* = 0.009	0.35
APC (20–30 s) [W]	1344.76 ± 396.80	1644.14 ± 418.60*	−299.38	*P* = 0.03	0.73	1208.68 ± 484.42	1435.23 ± 426.23*	−226.55	*P* = 0.05	0.50
APD (0–30 s) [W]	1485.45 ± 367.07	1787.64 ± 328.41	−302.19	*P* = 0.62	0.87	1598.76 ± 295.55	1617.70 ± 495.15	−18.94	*P* = 0.31	0.04
AFA (0–10 s) [N]	1431.94 ± 414.74	1612.29 ± 227.22	−180.35	*P* = 0.35	0.54	1361.79 ± 229.74*	1347.92 ± 284.64	13.87	*P* = 0.00	0.05
AFB (10–20 s) [N]	1271.80 ± 431.38	1462.61 ± 276.40	−190.81	*P* = 0.81	0.53	1385.23 ± 277.69	1382.43 ± 240.54	2.8	*P* = 0.30	0.01
AFC (20–30 s) [N]	1261.88 ± 313.95	1479.93 ± 256.00	−218.05	*P* = 0.70	0.76	1085.23 ± 255.56	1306.29 ± 208.73*	−221.06	*P* = 0.01	0.95
AFD (0–30 s) [N]	1321.80 ± 350.04	1517.50 ± 207.27	−195.7	*P* = 0.62	0.68	1265.41 ± 231.55	1367.93 ± 227.23*	−102.52	*P* = 0.002	0.45

*P < 0.05; **md**, mean differences, **Cohen d** effect size where ≤ 0.2 = small, ≤ 0.5 = medium, and ≤ 0.8 = large.

According to Table 4, there was a statistically significant difference between the deaf basketball and deaf volleyball groups in favor of the deaf volleyball group in terms of JHTOVA, JHTOVB, JHTOVC, JHTOVD, JHFTA, and JHFTB parameters. A statistically significant difference was found in favor of deaf basketball players in the JHTOV parameter. A statistically significant difference was revealed between the non-deaf basketball and non-deaf volleyball groups in favor of the non-deaf volleyball group in terms of JHTOVA, JHTOVB, JHTOVC, JHTOVD, JHFTA, JHFTB, JHFTC, and JHFTD parameters. There was a statistically significant difference in favor of the non-deaf basketball group in the JHTOV parameter.

**Table 4:** Comparison of jump height from take off velocity and jump height from flight time of deaf basketball/volleyball and non-deaf basketball/volleyball players.

**Parameter's**	**Deafness basketball (n:11)**	**Deafness volleyball (n:12)**	***md***	***p***	***d***	**Basketball (n:14)**	**Volleyball (n:14)**	***md***	***p***	***d***
	**X ±SS**	**X ±SS**				**X ±SS**	**X ±SS**			
JHTOV (total jumps over 30 s)	21.04 ± 4.54*	17.42 ± 4.66	3.62	*P* = 0.05	0.79	23.36 ± 4.78*	19.71 ± 3.60	3.65	*P* = 0.03	0.86
JHTOVA (0–10 s) [m]	0.24 ± 0.09	0.32 ± 0.09*	−0.08	*P* = 0.02	0.89	0.28 ± 0.08	0.35 ± 0.06*	−0.07	*P* = 0.02	0.99
JHTOVB (10–20 s) [m]	0.21 ± 0.05	0.27 ± 0.04*	−0.06	*P* = 0.02	1.32	0.23 ± 0.07	0.32 ± 0.08*	−0.09	*P* = 0.008	1.18
JHTOVC (20–30 s) [m]	0.16 ± 0.06	0.26 ± 0.16*	−0.1	*P* = 0.00	0.83	0.20 ± 0.05	0.31 ± 0.10**	−0.11	*P* = 0.000	1.39
JHTOVD (0–30 s) [m]	0.21 ± 0.04	0.26 ± 0.04*	−0.05	*P* = 0.02	1.25	0.24 ± 0.06	0.33 ± 0.07**	−0.09	*P* = 0.000	1.38
JHFTA (0–10 s) [m]	0.21 ± 0.06	0.26 ± 0.07*	−0.05	*P* = 0.03	0.78	0.23 ± 0.05	0.29 ± 0.04*	−0.06	*P* = 0.000	1.32
JHFTB (10–20 s) [m]	0.19 ± 0.05	0.25 ± 0.05*	−0.06	*P* = 0.00	1.20	0.20 ± 0.05	0.28 ± 0.05**	−0.08	*P* = 0.000	1.6
JHFTC (20–30 s) [m]	0.17 ± 0.03	0.20 ± 0.06	−0.03	*P* = 0.23	0.63	0.18 ± 0.04	0.27 ± 0.05**	−0.09	*P* = 0.000	1.99
JHFTD (0–30 s) [m]	0.20 ± 0.03	0.23 ± 0.05	−0.03	*P* = 0.06	0.73	0.20 ± 0.03	0.29 ± 0.04**	−0.09	*P* = 0.000	2.54

*P < 0.05; **P < 0.001; **md**, mean differences, **Cohen d** effect size where ≤ 0.2 = small, ≤ 0.5 = medium, and ≤ 0.8 = large.

Upon examining Table 5, there was a statistically significant difference between the deaf basketball and deaf volleyball groups in favor of the deaf volleyball group in terms of CMAA, CMAC, and POAC parameters and there was a statistically significant difference in favor of the deaf basketball group in AVA and AVB parameters. A statistically significant difference was found between the non-deaf basketball and non-deaf volleyball groups in favor of the non-deaf volleyball group in POAA, AVA, AVC, and AVD parameters. No statistically significant difference was identified in other parameters (*p* > 0.05).

**Table 5:** Comparison of counter movement acceleration, push off acceleration and average velocity of deaf basketball/volleyball and non-deaf basketball/volleyball players.

**Parameter's**	**Deafness basketball (n:11)**	**Deafness volleyball (n:12)**	***md***	***p***	***d***	**Basketball (n:14)**	**Volleyball (n:14)**	***md***	***p***	***d***
	**X ±SS**	**X ±SS**				**X ±SS**	**X ±SS**			
CMAA (0–10 s) [m/s^∧^2]	6.80 ± 3.06	5.97 ± 2.57	0.83	*P* = 0.43	0.29	6.40 ± 2.11	5.23 ± 2.29	1.17	*P* = 0.67	0.53
CMAB (10–20 s) [m/s^∧^2]	6.24 ± 1.66	6.76 ± 2.46	−0.52	*P* = 0.57	0.25	6.12 ± 2.03	6.10 ± 2.53	0.02	*P* = 0.46	0.09
CMAC (20–30 s) [m/s^∧^2]	6.47 ± 1.77	5.28 ± 1.62	1.19	*P* = 0.18	0.70	6.07 ± 2.48	5.39 ± 2.32	0.68	*P* = 0.40	0.28
CMAD (0–30 s) [m/s^∧^2]	6.22 ± 1.98	5.72 ± 2.40	0.5	*P* = 0.85	0.23	6.02 ± 2.24	5.76 ± 2.19	0.26	*P* = 0.73	0.12
POAA (0–10 s) [m/s^∧^2]	7.26 ± 2.16	7.09 ± 1.89	0.17	*P* = 0.84	0.08	7.16 ± 1.44	5.81 ± 2.56*	1.35	*P* = 0.08	0.65
POAB (10–20 s) [m/s^∧^2]	6.28 ± 1.90	7.02 ± 2.26	−0.74	*P* = 0.35	0.35	6.02 ± 1.39	6.22 ± 2.01	−0.02	*P* = 0.78	0.11
POAC (20–30 s) [m/s^∧^2]	5.98 ± 1.13	4.80 ± 1.84	1.18	*P* = 0.09	0.77	6.00 ± 2.02	5.33 ± 1.38	0.67	*P* = 0.28	0.39
POAD (0–30 s) [m/s^∧^2]	6.01 ± 1.47	6.30 ± 1.37	−0.29	*P* = 0.65	0.20	6.44 ± 1.63	6.05 ± 1.73	0.39	*P* = 0.50	0.23
AVA (0–10 s) [m/s^∧^2]	1.22 ± 0.23	1.38 ± 0.21	−0.16	*P* = 0.37	0.73	1.34 ± 0.22	0.97 ± 0.48*	0.37	*P* = 0.01	0.99
AVB (10–20 s) [m/s^∧^2]	1.12 ± 0.38*	1.37 ± 0.31	−0.25	*P* = 0.05	0.72	1.16 ± 0.42	1.03 ± 0.47	0.13	*P* = 0.57	0.29
AVC (20–30 s) [m/s^∧^2]	1.15 ± 0.21	1.12 ± 0.37	0.03	*P* = 0.85	0.10	1.20 ± 0.23	0.94 ± 0.39	0.26	*P* = 0.16	0.81
AVD (0–30 s) [m/s^∧^2]	1.18 ± 0.15	1.27 ± 0.17	−0.09	*P* = 0.48	0.56	1.24 ± 0.23	1.01 ± 0.42	0.23	*P* = 0.24	0.68

*P < 0.05; **md**, mean differences, **Cohen d** effect size where ≤ 0.2 = small, ≤ 0.5 = medium, and ≤ 0.8 = large.

As seen in Table 6, the APB parameter was statistically significant in favor of the deaf volleyball group and the AFC parameter was statistically significant in favor of the deaf basketball group. No statistically significant difference was found in the other parameters (*p* > 0.05). A statistically significant difference was determined between the non-deaf basketball and non-deaf volleyball groups in APA, APB, APC, APD, AFA, AFC, and AFD parameters (*p* < 0.05). This difference was found to be significant in favor of the non-deaf basketball group. Although there was a difference in favor of the basketball group in the AFB parameter, this difference was not found to be statistically significant (*p* > 0.05).

**Table 6:** Comparison of average power and force of deaf basketball/volleyball and non-deaf basketball/volleyball players.

**Parameter's**	**Deafness basketball (n:11)**	**Deafness volleyball (n:12)**	***md***	***p***	***d***	**Basketball (n:14)**	**Volleyball (n:14)**	***md***	***p***	***d***
	**X ±SS**	**X ±SS**				**X ±SS**	**X ±SS**			
APA (0–10 s) [W]	1618.96 ± 404.60	1787.17 ± 359.55	−168.21	*P* = 0.56	0.44	2028.14 ± 349.69*	1580.69 ± 609.01	447.45	*P* = 0.002	0.90
APB (10–20 s) [W]	1412.81 ± 543.25	1812.75 ± 420.87*	−399.94	*P* = 0.01	0.82	1742.47 ± 420.25*	1638.71 ± 560.35	103.76	*P* = 0.49	0.21
APC (20–30 s) [W]	1344.76 ± 396.80*	1208.68 ± 484.42	136.08	*P* = 0.03	0.31	1644.14 ± 418.60**	1435.23 ± 426.23	208.91	*P* = 0.000	0.49
APD (0–30 s) [W]	1485.45 ± 367.07	1598.76 ± 295.55*	−113.31	*P* = 0.05	0.34	1787.64 ± 328.41*	1617.70 ± 495.15	165.94	*P* = 0.12	0.40
AFA (0–10 s) [N]	1431.94 ± 414.74	1361.79 ± 229.74	70.15	*P* = 0.22	0.21	1612.29 ± 227.22**	1347.92 ± 284.64	264.37	*P* = 0.000	1.0
AFB (10–20 s) [N]	1271.80 ± 431.38	1385.23 ± 277.69	−113.43	*P* = 0.14	0.31	1462.61 ± 276.40	1382.43 ± 240.54	80.18	*P* = 0.39	0.31
AFC (20–30 s) [N]	1261.88 ± 313.95	1085.23 ± 255.56	176.65	*P* = 0.84	0.62	1479.93 ± 256.00*	1306.29 ± 208.73	176.64	*P* = 0.03	0.74
AFD (0–30 s) [N]	1321.80 ± 350.04	1265.41 ± 231.55	56.39	*P* = 0.48	0.19	1517.50 ± 207.27*	1367.93 ± 227.23	149.57	*P* = 0.03	0.69

*P < 0.05; **P < 0.001; **md**, mean differences, **Cohen d** effect size where ≤ 0.2 = small, ≤ 0.5 = medium, and ≤ 0.8 = large.

## Discussion

In the present study, RCMJ performances of (deaf/non-deaf) basketball and volleyball players were analyzed. The main highlight of our study is that it is the first study in the relevant literature, and it has added new reference points to the literature. When the results were evaluated, the jump performance parameters [jump height (Table 1), acceleration in the jump phase (Table 2), and force (Table 3)] of healthy basketball and volleyball players were found to be better compared to deaf athletes. When the deaf group and healthy athletes were compared within themselves, jump height (Table 4), acceleration in the jump phase (Table 5), and force (Table 6) were observed to be better in volleyball players. In the test protocol, the 30-s RCMJ was divided into three periods of 10 s, and the force produced during the jump, jump height, and acceleration in the jump phase were found to decrease linearly, and this decrease was sharper in deaf athletes. When analyzed in terms of sports branch, performance values of both deaf and non-deaf volleyball players were found to be better than basketball players in general.

Recent studies have scrutinized the biomechanical performance of the hip, knee, and ankle (extensor and flexor concentric, extensor and flexor eccentric) from the start of CMJ onward, by referring to multiple joints to produce a functional movement (Johnston et al., 2015; Kipp et al., 2016; Barker et al., 2018). Moreover, the jumping technique, the force produced during muscle contraction, and jump velocity have also been reported to be important factors (Floría and Harrison, 2013). They are important since basketball and volleyball players frequently use RCMJ during training and competition. It is clear that they are needed for performance when the jump action needs to be repeated successively. In this respect, their importance increases further for the performances of not only non-deaf athletes but also deaf athletes. However, there are many influencing factors (Gross et al., 2022). A study reported that there was no difference between deaf and non-deaf individuals in terms of gait ground reaction force (GRF) components, but the impulse observed in the deaf group disrupted the control of mediolateral body movement during gait, and this resulted from their less use of adductor muscles (Jafarnezhadgero et al., 2017). The 30-s test time we used in the study influenced the performance components in this respect. In parallel, there are studies in the literature stating that jump height adversely affects acute neuromuscular fatigue (Weinhandl et al., 2011; Lesinski et al., 2016). In our study, deaf and non-deaf athletes' mean 30-s and intermittent 10-s performance values in the RCMJ test decreased together with acute fatigue. When evaluated in this respect, deaf athletes' RCMJ performance values were lower (Table 1). In a parallel study, the postural oscillation values of deaf basketball and volleyball players were investigated. The study reported that volleyball players were better at many postural oscillation parameters, and particularly the dominant leg values were in favor of volleyball players (Makaraci et al., 2021a). It is obvious that the deformation in postural oscillation leads to more vestibular disorder in adductor muscles, causing the jump heights of deaf athletes to be lower than those of non-deaf athletes. A different study comparing Olympic deaf volleyball players and non-deaf volleyball players stated that non-deaf volleyball players had better CMJ, squat jump, and drop jump performances (Makaraci et al., 2021b). There are studies evaluating elite athletes' jump performances. In his study, Taylor revealed that volleyball players' jump heights (*P* = 0.01), double-leg (*P* = 0.03), and single-leg (*P* = 0.04) performances during training were statistically significant (Taylor et al., 2019). Another factor that cannot be overlooked in the RCMJ test performance is acute fatigue. It is observed that the performance of deaf athletes decreases as time and neuromuscular fatigue increase compared to non-deaf athletes. In this respect, the results of our study revealed the special training needs of deaf athletes.

The time taken to produce the highest amount of force in quick actions is quite short, and the change in the actual force-speed graph differs according to the isometric force produced (Zemková, 2016). Due to speed = displacement/time, stronger players seem to obtain positive changes in vertical speed (*p* = 0.088; d = 0.92) (Peng et al., 2019). It has been asserted that the extent of concentric performance during the jump movement is largely derived from the conditions of the eccentric phase (flexural velocity and magnitude) and is highly correlated (r = 0.90) with the eccentric force (Kristianslund et al., 2012). Therefore, eccentric force allows for increased negative acceleration and momentum and for the use of repetitive jump (i.e., series elastic component and stretch-reflex mechanisms), leading to faster acceleration and eventually better jump height (Peng et al., 2017). Our study elucidated that the acceleration of deaf athletes at each jump in RCMJ increased with the increase in time, and especially non-deaf volleyball players came into prominence (Tables 2, 5). Considering that it is branch-specific, this situation can be said to result from the more frequent use of RCMJ in terms of its biomechanics in volleyball. In addition, it was determined that the force produced in the RCMJ take-off phase (average 30 s and intermittent 10 s) was effective, but the force produced decreased as the time extended, especially in deaf athletes (Table 3). Upon reviewing the literature, it is seen that many studies have focused on the force produced by jump parameters such as CMJ, Drop Jump, and Squat Jump in the lower extremity (Gathercole et al., 2015; Claudino et al., 2016; Makaraci et al., 2021b). The flexion/extension of the lower extremity and velocity changes at the time of jump, the forces between the feet and the ground, and the forces created at the time of CMJ influence the potential upward propelling energy of the athlete (Rice et al., 2017). In the phase of switching to full extension of the body just before the feet are taken off the ground, the potential energy created by the strong eccentric contraction of the flexors and the kinetic energy created by the concentric contraction of the extensor muscles help the body be pulled upward (Nikolaidis et al., 2015). Studies set forth the relationship between the force produced in the lower extremity and jump performance (r = 0.64–0.74) (Dawes and Spiteri, 2016; Pérez-Castilla et al., 2021). Another study supporting this reported a positive correlation between the force produced and the maximum power of a strong jump and landing ability in basketball and volleyball players (Suchomel et al., 2016). It is clear that this difference is especially related to anaerobic power and affects the force. Negative improvement in acceleration was an important factor that we found increased with acute muscle fatigue. As the performance of deaf athletes increases with time and neuromuscular fatigue, a decrease in jump speed and strength is observed compared to more non-deaf athletes. The results of our study, we think that the acceleration in the jump speed of the deaf athletes occurs due to the loss of strength.

Our study has limitations and strengths. Our participants were male basketball and volleyball players who received chronic training and had adequate exercise experience. Therefore, our results may not be valid for female or recreational athletes since they may give different physiological responses to these mechanical loads associated with jumping. Moreover, the athletes were at the preparation stage. Attention should be paid when comparing the results of our study with other athletes who may be at different training and physical preparation levels. Furthermore, a cross-sectional design was used in the study. Longitudinal studies should be conducted to reveal whether such relative jump parameters can ensure different training adaptations. It is deemed necessary to conduct further studies on relative and absolute jump parameters and their effects on the magnitude of the load, such as muscle, joint, connective tissue, muscle activation, and Hoffmann's reflex. Especially the fact that more athletes were not analyzed to determine the differences in the different playing positions of the players. The strongest aspect of our study is that this sample group is at an elite level and it also reveals new suggestions for the field in terms of the performance components examined.

This is the first study to investigate the number of jumps and jump height, the force produced, acceleration at the time of jump, and jump velocity during 30 s in deaf and non-deaf basketball and volleyball players within the scope of individual RCMJ test. The present study provided new information on the RCMJ performances of deaf and non-deaf athletes. The concept will pioneer the information similar to anaerobic scaling, based on the athlete's continuation of his maximum jump for 30 s and creating fatigue in the lower extremity. Relying on the biomechanical changes observed in this study, our findings indicate that there is a decrease in the number of jumps and jump heights, the force produced, the acceleration at the time of jump, and the jump velocity in all athletes, which particularly affects deaf athletes more. Trainers and other practitioners should consider measuring their athletes' performances before training or applying repetitive CMJ exercises in training to adjust the number of jumps and jump heights, the force produced, the acceleration at the time of jump, and jump velocity more effectively.

## Data availability statement

The original contributions presented in the study are included in the article/supplementary material, further inquiries can be directed to the corresponding author.

## Ethics statement

The studies involving human participants were reviewed and approved by Clinical Ethics Committee of Karamanoglu Mehmetbey University. The patients/participants provided their written informed consent to participate in this study.

## Author contributions

RS: idea, concept, design, control, supervision, and writing the article. AU: data collection and processing, references, and fundings. ÖÖ: analysis and interpretation, literature review, and critical review. AU and ÖP: materials. All authors contributed to the article and approved the submitted version.

## Conflict of interest

The authors declare that the research was conducted in the absence of any commercial or financial relationships that could be construed as a potential conflict of interest.

## Publisher's note

All claims expressed in this article are solely those of the authors and do not necessarily represent those of their affiliated organizations, or those of the publisher, the editors and the reviewers. Any product that may be evaluated in this article, or claim that may be made by its manufacturer, is not guaranteed or endorsed by the publisher.

## Abbreviations

ROM: Range of Motion
CMJ: Counter Movement Jump
RCMJ: Repetitive Counter Movement Jump
JHTOV: Jump Height From Take off Velocity
JHFT: Jump Height From Fkight Time
CMA: Counter Movement Acceleration
POA: Push off Acceleration
AV: Average Velocity
AP: Average Power
AF: Average Force
GRF: Gait Ground Reaction Force.
